# Supplementary material for: ATR/Mec1 prevents lethal meiotic recombination initiation on partially replicated chromosomes in budding yeast
Source: eLife. 2013 Oct 1;2:e00844. doi: 10.7554/eLife.00844 (PMC3787542; doi:10.7554/eLife.00844)
Supplement: Supplementary file 1. — Genotypes of yeast strains used in this study. DOI: http://dx.doi.org/10.7554/eLife.00844.016 [file elife00844s001.docx]

**Blitzblau, Supplementary File 1**

| **Strain No.** | **Relevant genotype** | **Reference** |
| --- | --- | --- |
| H118 =  NKY1455 | MATa, ho::LYS2, lys2, leu2::hisG, his4X::LEU2-URA3, ura3,  MATalpha, ho::LYS2, lys2, leu2::hisG, his4B::LEU2, ura3,  arg4-Bgl2, dmc1Δ::ARG4  arg4-nsp, dmc1Δ::ARG4 | Bishop, et al., 1992 |
| H154 =  A10912 | MATa, ho::LYS2, lys2, ura3, leu2::hisG, TRP, HIS  MATalpha, ho::LYS2, lys2, ura3, leu2::hisG, TRP, his3::hisG,  his4X::LEU2-(Bam)-URA3, arg4-Nsp  his4B::LEU2, arg4-Bgl II  cdc6::pSCC1-3HA-CDC6::KanMX6  cdc6::pSCC1-3HA-CDC6::KanMX6 | Hochwagen, et al., 2005 |
| H155 = A11265 | MATa, ho::LYS2, lys2, ura3, leu2::hisG, TRP, HIS  MATalpha, ho::LYS2, lys2, ura3, leu2::hisG, TRP, his3::hisG,  his4X::LEU2-(Bam)-URA3, rad50S::URA3  his4B::LEU2, arg4-Bgl II, rad50S::URA3  cdc6::pSCC1-3HA-CDC6::KanMX6,  cdc6::pSCC1-3HA-CDC6::KanMX6, | Hochwagen, et al., 2005 |
| H156 = A11675 | MATa, ho::LYS2, lys2, ura3, leu2::hisG, TRP, HIS  MATalpha, ho::LYS2, lys2, ura3, leu2::hisG, TRP, his3::hisG,  his4X::LEU2-(Bam)-URA3, rad50S::URA3  his4B::LEU2, arg4-Bgl II, rad50S::URA3 | Hochwagen, et al., 2005 |
| H385 | MATa, ho::LYS2, ura3, his3::hisG, trp1::hisG  MATalpha, ho::LYS2, ura3, his3::hisG, trp1::hisG  lys2::TetOx240:URA3, leu2::LEU2 tetR-GFP  lys2::TetOx240:URA3, leu2::LEU2 tetR-GFP  ndt80Δ::PSTE5:URA3  ndt80Δ::PSTE5:URA3 | This study |
| H386 | MATa, ho::LYS2, ura3, his3::hisG, trp1::hisG  MATalpha, ho::LYS2, ura3, his3::hisG, trp1::hisG  lys2::TetOx240:URA3, leu2::LEU2 tetR-GFP  lys2::TetOx240:URA3, leu2::LEU2 tetR-GFP  ndt80Δ::PSTE5:URA3, cdc6::pSCC1-3HA-CDC6::KanMX6  ndt80Δ::PSTE5:URA3, cdc6::pSCC1-3HA-CDC6::KanMX6 | This study |
| H1785 = SB1505 | MATa, ho::LYS2, lys2, ura3, leu2::hisG. his3::hisG,  trp1::hisG, flo8Δ::KanMX | Blitzblau, et al., 2012 |
| H2087 | MATa, ho::LYS2, lys2, ura3, leu2::hisG, trp1::hisG, his3::hisG,  MATalpha, ho::LYS2, lys2, ura3, leu2::hisG, trp1::hisG, his3::hisG  his4B::LEU2, arg4-Bgl II, SPO11-18MYC::TRP1  his4B::LEU2, arg4-Bgl II, SPO11-18MYC::TRP1 | This study |
| H2544=  A4224 | MATalpha, ho::LYS2, lys2, HIS4, leu2::hisG, trp1::hisG, ura3,  MATa, ho::LYS2, lys2, his4X, leu2::hisG, TRP1, ura3,  cln3Δ::LEU2  cln3Δ::LEU2 | Blitzblau, et al., 2012 |
| H2560 | MATa, ho::LYS2, lys2, leu2::hisG, ura3, trp1::hisG, his3::hisG,  MATalpha, ho::LYS2, lys2, leu2::hisG, ura3, trp1::hisG, his3::hisG,  mec1-1, sml1Δ::KanMX  mec1-1, sml1Δ::KanMX | This study |
| H2591 | MATa, ho::LYS2, lys2, ura3, leu2::hisG, his3::hisG, trp1::hisG  MATalpha, ho::LYS2, lys2, ura3, leu2::hisG, his3::hisG, trp1::hisG  sml1Δ::KanMX, rad53Δ::TRP1  sml1Δ::KanMX, rad53Δ::TRP1 | This study |
| H2636 | MATa, ho::hisG, leu2::hisG, ura3(Δsma-pst::hisG),  MATalpha, ho::hisG, leu2::hisG, ura3(Δsma-pst::hisG),  HIS4::LEU2-(NBam;ori)  his4X::LEU2-(NgoMIV)-URA3 | This study |
| H2655 | MATa, ho::hisG, lys2, leu2::hisG, ura3(Δsma-pst::hisG), his3::hisG,  MATalpha, ho::hisG(?), LYS2, leu2::hisG, ura3(Δsma-pst::hisG), HIS3  his4X::LEU2-(NgoMIV)-URA3, cdc6::pSCC1-3HA-CDC6::KanMX6  HIS4::LEU2-(NBam;ori), cdc6::pSCC1-3HA-CDC6::KanMX6 | This study |
| H2775 | MATa, ho::LYS2, lys2, his3::hisG, leu2::hisG, ura3, trp1::hisG,  MATalpha, ho::LYS2, lys2, his3::hisG, leu2::hisG, ura3, trp1::hisG,  cln3Δ::LEU2, rad50S::URA3  cln3Δ::LEU2, rad50S::URA3 | This study |
| H2776 | MATa, ho::LYS2, lys2, his3::hisG, leu2::hisG, ura3, trp1::hisG,  MATalpha, ho::LYS2, lys2, his3::hisG, leu2::hisG, ura3, trp1::hisG,  cln3Δ::LEU2, cdc6::pSCC1-3HA-CDC6::KanMX6, rad50S::URA3  cln3Δ::LEU2, cdc6::pSCC1-3HA-CDC6::KanMX6, rad50S::URA3 | This study |
| H3554 | MATa, ho::LYS2, lys2, leu2::hisG, ura3, trp1::hisG, his3::hisG,  MATalpha, ho::LYS2, lys2, ura3, leu2::hisG, his3::hisG, trp1::hisG  sml1Δ::KanMX  sml1Δ::KanMX, mec1-1 (het), pph3Δ::LEU2 (het) | This study |
| H3598 | MATalpha, ho::LYS(?), lys2(?), leu2::hisG, ura3(Δsma-pst::hisG),  MATa, ho::hisG, lys2, leu2::hisG, ura3(Δsma-pst::hisG),  his3::hisG, TRP1, HIS4::LEU2-(NBam;ori),  his3::hisG, TRP1, his4X::LEU2-(NgoMIV)-URA3,  cdc6::pSCC1-3HA-CDC6::KanMX6, spo11-HA3-His6::KanMX4  cdc6::pSCC1-3HA-CDC6::KanMX6, spo11-Y135F::KanMX4 | This study |
| H3682 | MATa, ho::LYS2, lys2, ura3, his3::hisG,  MATalpha, ho::LYS2, ura3, his3::hisG,  lys2::TetOx240:URA3, leu2::LEU2 tetR-GFP, ndt80Δ::Pste5:URA3  lys2::TetOx240:URA3, leu2::LEU2 tetR-GFP, ndt80Δ::Pste5:URA3  cdc6::pSCC1-3HA-CDC6::KanMX6, spo11Δ::URA3  cdc6::pSCC1-3HA-CDC6::KanMX6, spo11Δ::URA3 | This study |
| H3753 | MATa, ho::LYS2, ura3, his3::hisG, trp1::hisG, leu2::hisG,  MATalpha, ho::LYS2, ura3, his3::hisG, trp1::hisG,  leu2::pURA3-TetR-GFP::LEU2 (het), CENV::TetOx224::HIS3 (het)  cdc6::pSCC1-3HA-CDC6::KanMX6, spo11Δ::TRP1 (het)  cdc6::pSCC1-3HA-CDC6::KanMX6 | This study |
| H3754 | MATa, ho::LYS2, ura3, his3::hisG, trp1::hisG, leu2::hisG,  MATalpha, ho::LYS2, ura3, his3::hisG, trp1::hisG,  leu2::pURA3-TetR-GFP::LEU2 (het), CENV::TetOx224::HIS3 (het)  cdc6::pSCC1-3HA-CDC6::KanMX6, spo11Δ::TRP1  cdc6::pSCC1-3HA-CDC6::KanMX6, spo11Δ::TRP1 | This study |
| H3755 | MATa, ho::LYS2, ura3, his3::hisG, trp1::hisG, leu2::hisG,  MATalpha, ho::LYS2, lys2, ura3, his3::hisG, trp1::hisG  leu2::pURA3-TetR-GFP::LEU2 (het), CENV::TetOx224::HIS3 (het)  cdc6::pSCC1-3HA-CDC6::KanMX6 (het), spo11Δ::TRP1 (het) | This study |
| H3756 | MATa, ho::LYS2, lys2?, ura3, leu2, his3::hisG, trp1::hisG  MATalpha, ho::LYS2, ura3, leu2::hisG, his3::hisG, trp1::hisG,  promURA3::tetR:: GFP-LEU2, (het), TelV::tetOx224::URA3 (het)  cdc6::pSCC1-3HA-CDC6::KanMX6, spo11Δ::TRP1 (het)  cdc6::pSCC1-3HA-CDC6::KanMX6 | This study |
| H3757 | MATa, ho::LYS2, lys2?, ura3, his3::hisG, trp1::hisG  MATalpha, ho::LYS2, ura3, leu2::hisG, his3::hisG, trp1::hisG,  promURA3::tetR:: GFP-LEU2 (het), TelV::tetOx224::URA3 (het)  spo11Δ::TRP1, cdc6::pSCC1-3HA-CDC6::KanMX6  spo11Δ::TRP1, cdc6::pSCC1-3HA-CDC6::KanMX6 | This study |
| H3758 | MATa, ho::LYS2, lys2?, ura3, his3::hisG, trp1::hisG  MATalpha, ho::LYS2, lys2, ura3, leu2::hisG, his3::hisG, trp1::hisG promURA3::tetR:: GFP-LEU2 (het), TelV::tetOx224::URA3 (het)  spo11Δ::TRP1 (het), cdc6::pSCC1-3HA-CDC6::KanMX6 (het) | This study |
| H3803 | MATa, ho::LYS2, lys2, his4X, ura3, TRP,  MATalpha, ho::LYS2, ura3, leu2::hisG, his3::hisG, trp1::hisG,  lys2::TetOx240:URA3 (het), leu2::LEU2 tetR-GFP (het)  cdc6::pSCC1-3HA-CDC6::KanMX6, spo11Δ::URA3 (het)  cdc6::pSCC1-3HA-CDC6::KanMX6 | This study |
| H3804 | MATa, ho::LYS2, lys2, his4X, ura3, TRP,  MATalpha, ho::LYS2, ura3, leu2::hisG, his3::hisG, trp1::hisG,  lys2::TetOx240:URA3 (het), leu2::LEU2 tetR-GFP (het)  cdc6::pSCC1-3HA-CDC6::KanMX6, spo11Δ::URA3  cdc6::pSCC1-3HA-CDC6::KanMX6, spo11Δ::TRP1 | This study |
| H3805 | MATa, ho::LYS2, lys2, his4X, ura3, TRP,  MATalpha, ho::LYS2, lys2, ura3, leu2::hisG, his3::hisG, trp1::hisG lys2::TetOx240:URA3 (het), leu2::LEU2 tetR-GFP (het)  cdc6::pSCC1-3HA-CDC6::KanMX6 (het), spo11Δ::URA3 (het) | This study |
| H4226 | MATa, ho::hisG, lys2, leu2::hisG, ura3(Δsma-pst::hisG),  MATalpha, ho::LYS2, LYS2, leu2::hisG, ura3(Δsma-pst::hisG),  his4X::LEU2-(NgoMIV)-URA3, rad50S::URA3  HIS4::LEU2-(NBam;ori), rad50S::URA3 | This study |
| H4557 | MATa, ho::LYS2, lys2, leu2::hisG, his3::hisG(?) ura3, arg4-Bgl2(?),  MATalpha, ho::LYS2, lys2, leu2::hisG, his3::hisG(?), ura3, arg4-nsp,  his4B::LEU2, dmc1Δ::ARG4, mec1-1, sml1Δ::KanMX  his4X::LEU2-URA3, dmc1Δ::ARG4, mec1-1, sml1Δ::KanMX | This study |
| H4585 | MATa, ho::LYS2, lys2, ura3, leu2::hisG, his3::hisG, trp1::hisG,  MATalpha, ho::LYS2, lys2, ura3, leu2::hisG, his3::hisG, trp1::hisG,  mer2(rec107)::MER2-5myc, REC8-3HA-URA3  mer2(rec107)::MER2-5myc, REC8-3HA-URA3 | This study |
| H4618 | MATa, ho::LYS2, lys2, leu2::hisG, his3::hisG(?) ura3, arg4-Bgl2(?),  MATalpha, ho::LYS2, lys2, leu2::hisG, his3::hisG(?), ura3, arg4-nsp(?)  his4B::LEU2, dmc1Δ::ARG4, sml1Δ::KanMX  his4X::LEU2-URA3, dmc1Δ::ARG4, sml1Δ::KanMX | This study |
| H4695 | MATalpha, ho::LYS2, lys2, ura3, LEU2, his3::hisG or his4X,  MATa, ho::LYS2, lys2, ura3, leu2::hisG, HIS3, HIS4,  mer2(rec107)::MER2-5myc, REC8-3HA-URA3  mer2(rec107)::MER2-5myc, REC8-3HA-URA3 | This study |
| H4849 | MATa, ho::LYS2, lys2, ura3, TRP1, ,  MATalpha, ho::LYS2, lys2, ura3, trp1::hisG,  sml1Δ::KanMX, tel1Δ::HIS3, rad50S::URA3, mec1-1  sml1Δ::KanMX, tel1Δ::HIS3, rad50S::URA3 | This study |
| H4850 | MATa, ho::LYS2, lys2, leu2::hisG, ura3, TRP1, his3::hisG,  MATalpha, ho::LYS2, lys2, LEU2, ura3, trp1::hisG, HIS3,  sml1Δ::KanMX, rad50S::URA3, mec1-1 (het), tel1Δ::HIS3 (het)  sml1Δ::KanMX, rad50S::URA3 | This study |
| H4851 | MATa, ho::LYS2, lys2, ura3, TRP1,  MATalpha, ho::LYS2, lys2, ura3, trp1::hisG,  mec1-1, sml1Δ::KanMX, rad50S::URA3, tel1Δ::HIS3 (het)  mec1-1, sml1Δ::KanMX, rad50S::URA3 | This study |
| H4853 | MATa, ho::LYS2, lys2, leu2::hisG, ura3, TRP1,  MATalpha, ho::LYS2, lys2, LEU2, ura3, trp1::hisG,  mec1-1, sml1Δ::KanMX, tel1Δ::HIS3, rad50S::URA3  mec1-1, sml1Δ::KanMX, tel1Δ::HIS3, rad50S::URA3 | This study |
| H4883 | MATa, ho::LYS2, lys2, ura3, leu2::hisG, his3::hisG, trp1::hisG  MATalpha, ho::LYS2, lys2, ura3, leu2::hisG, his3::hisG, trp1::hisG  rad50S::URA3, mcm5::bob1-1-HphMX4, dbf4Δ::TRP1,  rad50S::URA3, mcm5::bob1-1-HphMX4, dbf4Δ::TRP1,  [dbf4-T163A::LEU2]  [dbf4-T163A::LEU2] | This study |
| H4890 | MATa, ho::LYS2, TRP1, his3::hisG, ura3, lys2, leu2::hisG,  MATalpha, ho::LYS2, trp1::hisG, his3::hisG, URA3, lys2, LEU2,  REC114-13MYC::HIS3  REC114-13MYC::HIS3 | This study |
| H4898 | MATa, ho::LYS2, lys2, leu2::hisG, ura3, TRP1, his3::hisG,  MATalpha, ho::LYS2, lys2, LEU2, ura3, trp1::hisG, HIS3,  sml1Δ::KanMX, rad50S::URA3  sml1Δ::KanMX, rad50S::URA3 | Blitzblau et al. 2012 |
| H4932 | MATa, ho::LYS2, lys2, ura3, leu2::hisG, HIS3, trp1::hisG,  MATalpha, ho::LYS2, lys2, ura3, LEU2, his3::hisG, trp1::hisG,  mec1Δ::TRP1, rad53Δ::TRP1, sml1Δ::KanMX, rad50S::URA3  mec1Δ::TRP1, rad53Δ::TRP1, sml1Δ::KanMX, rad50S::URA3 | This study |
| H4935 | MATa, ho::LYS2, lys2, ura3, LEU2, his3::hisG, trp1::hisG,  MATalpha, ho::LYS2, lys2, ura3, leu2::hisG, HIS3, trp1::hisG,  mec1Δ::TRP1, sml1Δ::KanMX, rad50S::URA3  mec1Δ::TRP1, sml1Δ::KanMX, rad50S::URA3 | This study |
| H4969 | MATa, ho::LYS2, lys2, ura3, leu2::hisG, HIS3, trp1::hisG,  MATalpha, ho::LYS2, lys2, ura3, LEU2, his3::hisG, trp1::hisG,  sml1Δ::KanMX, rad53Δ::TRP1, rad50S::URA3  sml1Δ::KanMX, rad53Δ::TRP1, rad50S::URA3 | This study |
| H5076 | MATa, ho::LYS2, ura3, leu2::hisG, his3::hisG, trp1::hisG,  MATalpha, ho::LYS2, ura3, leu2::hisG, his3::hisG, trp1::hisG,  clb5Δ::KanMX, clb6Δ::TRP1, mer2(rec107)::MER2-5myc  clb5Δ::KanMX, clb6Δ::TRP1, mer2(rec107)::MER2-5myc | This study |
| H5079 | MATa, ho::LYS2, ura3, leu2::hisG, his3::hisG, his4X, trp1::hisG,  MATalpha, ho::LYS2, ura3, leu2::hisG, his3::hisG, trp1::hisG,  ndt80Δ::TRP1, spo11-Y135F-HA-URA3, mer2(rec107)::MER2-5myc  ndt80Δ::TRP1, spo11-Y135F-HA-URA3, mer2(rec107)::MER2-5myc | This study |
| H5082 | MATa, ho::LYS2, ura3, leu2::hisG, his3::hisG, his4X, trp1::hisG,  MATalpha, ho::LYS2, ura3, leu2::hisG, his3::hisG, trp1::hisG,  ndt80Δ::TRP1, spo11-Y135F-HA-URA3, SAE2(COM1)-13myc::HIS3  ndt80Δ::TRP1, spo11-Y135F-HA-URA3, SAE2(COM1)-13myc::HIS3 | This study |
| H5085 | MATa, ho::LYS2, ura3, leu2::hisG, his3::hisG, trp1::hisG,  MATalpha, ho::LYS2, ura3, leu2::hisG, his3::hisG, his4X, trp1::hisG,  ndt80Δ::TRP1, spo11-Y135F-HA-URA3, MRE11-13myc::HIS3  ndt80Δ::TRP1, spo11-Y135F-HA-URA3, MRE11-13myc::HIS3 | This study |
| H5088 | MATa, ho::LYS2, ura3, leu2::hisG, his3::hisG, trp1::hisG,  MATalpha, ho::LYS2, ura3, leu2::hisG, his3::hisG, his4X, trp1::hisG,  ndt80Δ::TRP1, spo11-Y135F-HA-URA3, REC104-13myc::HIS3  ndt80Δ::TRP1, spo11-Y135F-HA-URA3, REC104-13myc::HIS3 | This study |
| H5092 | MATa, ho::LYS2, ura3, leu2::hisG, his3::hisG, trp1::hisG,  MATalpha, ho::LYS2, ura3, leu2::hisG, his3::hisG, his4X, trp1::hisG,  ndt80Δ::TRP1, spo11-Y135F-HA-URA3, REC114-13myc::HIS3  ndt80Δ::TRP1, spo11-Y135F-HA-URA3, REC114-13myc::HIS3 | This study |
| H5095 | MATa, ho::LYS2, ura3, leu2::hisG, his3::hisG, trp1::hisG,  MATalpha, ho::LYS2, ura3, leu2::hisG, his3::hisG, trp1::hisG,  ndt80Δ::TRP1, spo11-Y135F-HA-URA3, MEI4-13myc::HIS3  ndt80Δ::TRP1, spo11-Y135F-HA-URA3, MEI4-13myc::HIS3 | This study |
| H5098 | MATa, ho::LYS2, ura3, leu2::hisG, his3::hisG, trp1::hisG,  MATalpha, ho::LYS2, ura3, leu2::hisG, his3::hisG, his4X, trp1::hisG,  ndt80Δ::TRP1, spo11-Y135F-HA-URA3, XRS2-13myc::HIS3  ndt80Δ::TRP1, spo11-Y135F-HA-URA3, XRS2-13myc::HIS3 | This study |
| H5127 | MATa, ho::LYS2, lys2, ura3, leu2::hisG, his3::hisG, trp1::hisG,  MATalpha, ho::LYS2, lys2, ura3, leu2::hisG, his3::hisG, trp1::hisG,  sml1Δ::KanMX, rad53Δ::TRP1, mer2(rec107)::MER2-5myc  sml1Δ::KanMX, rad53Δ::TRP1, mer2(rec107)::MER2-5myc | This study |
| H5154 | MATa, ho::LYS2, ura3, leu2::hisG, his3::hisG, trp1::hisG,  MATalpha, ho::LYS2, ura3, leu2::hisG, his3::hisG, trp1::hisG,  ndt80Δ::TRP1, spo11-Y135F-HA-URA3, SKI8-13MYC::HIS3  ndt80Δ::TRP1, spo11-Y135F-HA-URA3, SKI8-13MYC::HIS3 | This study |
| H5157 | MATa, ho::LYS2, lys2, ura3, leu2::hisG, HIS3, trp1::hisG,  MATalpha, ho::LYS2, lys2, ura3, LEU2, his3::hisG, trp1::hisG,  sml1Δ::KanMX, mer2(rec107)::MER2-5myc, mec1Δ::TRP1 (het)  sml1Δ::KanMX, mer2(rec107)::MER2-5myc | This study |
| H5220 | MATa, ho::LYS2, lys2, ura3, leu2::hisG, HIS3, trp1::hisG,  MATalpha, ho::LYS2, lys2, ura3, LEU2, his3::hisG, trp1::hisG,  mec1Δ::TRP1, sml1Δ::KanMX, mer2(rec107)::MER2-5myc  mec1Δ::TRP1, sml1Δ::KanMX, mer2(rec107)::MER2-5myc  rad50S::URA3 (het) | This study |
| H5227 | MATa, ho::LYS2, lys2, leu2::hisG, ura3, trp1::hisG, his3::hisG,  MATalpha, ho::LYS2, lys2, leu2::hisG, ura3, trp1::hisG, his3::hisG,  ndt80Δ::TRP1, spo11-Y135F-HA-URA3, mec1-1, sml1Δ::KanMX  ndt80Δ::TRP1, spo11-Y135F-HA-URA3, mec1-1, sml1Δ::KanMX | This study |
| H5230 | MATa, ho::LYS2, lys2, leu2::hisG, ura3, trp1::hisG, his3::hisG,  MATalpha, ho::LYS2, lys2, leu2::hisG, ura3, trp1::hisG, his3::hisG,  ndt80Δ::TRP1, spo11-Y135F-HA-URA3, rad53Δ::TRP1,  ndt80Δ::TRP1, spo11-Y135F-HA-URA3, rad53Δ::TRP1,  sml1Δ::KanMX  sml1Δ::KanMX | This study |
| H5233 | MATa, ho::LYS2, lys2, leu2::hisG, ura3, trp1::hisG, his3::hisG,  MATalpha, ho::LYS2, lys2, leu2::hisG, ura3, trp1::hisG, his3::hisG,  ndt80Δ::TRP1, spo11-Y135F-HA-URA3, sml1Δ::KanMX  ndt80Δ::TRP1, spo11-Y135F-HA-URA3, sml1Δ::KanMX | This study |
| H5241 | MATa, ho::LYS2, lys2, ura3, leu2::hisG, his3::hisG, trp1::hisG,  MATalpha, ho::LYS2, lys2, ura3, leu2::hisG, his3::hisG, trp1::hisG,  sml1Δ::KanMX, rad53Δ::TRP1, mek1Δ::LEU2, chk1Δ::HIS3,  sml1Δ::KanMX, rad53Δ::TRP1, mek1Δ::LEU2, chk1Δ::HIS3,  rad50S::URA3  rad50S::URA3 | This study |
| H5249 | MATa, ho::LYS2, ura3, leu2::hisG, his3::hisG, trp1::hisG,  MATalpha, ho::LYS2, ura3, leu2::hisG, his3::hisG, trp1::hisG,  ndt80Δ::TRP1, spo11-Y135F-HA-URA3, REC102-13MYC-HIS3MX6  ndt80Δ::TRP1, spo11-Y135F-HA-URA3, REC102-13MYC-HIS3MX6 | This study |
| H5547 | MATa, ho::LYS2, TRP, his3::hisG, ura3, LEU2  MATalpha, ho::LYS2, trp1::hisG, his3::hisG, URA3, leu2::hisG  MRE11-13MYC::HIS3  MRE11-13MYC::HIS3 | This study |
| H5603 | MATa, ho::LYS2, lys2, ura3, leu2::hisG, trp1::hisG  MATalpha, ho::LYS2, lys2, ura3, leu2::hisG, HIS3, trp1::hisG  dbf4Δ::TRP1, Pdbf4::Pdbf4-dbf4-m25-LEU2, rad50S::URA3  dbf4Δ::TRP1, Pdbf4::Pdbf4-dbf4-m25-LEU2, rad50S::URA3 | This study |
| H5776 | MATa, ho::LYS2, lys2, HIS3, leu2::hisG, ura3, trp1::hisG,  MATalpha, ho::LYS2, lys2, his3::hisG, leu2::hisG, ura3, trp1::hisG,  rad50S::URA3, mrc1Δ::TRP, rad9Δ::LEU2, sml1Δ::KanMX  rad50S::URA3, mrc1Δ::TRP, rad9Δ::LEU2, sml1Δ::KanMX | This study |
| H5884 | MATa, ho::hisG, lys2, leu2::hisG, ura3(Δsma-pst::hisG),  MATalpha, ho::LYS2, lys2, leu2::hisG, ura3(Δsma-pst::hisG),  HIS3, trp1::hisG,  his3::hisG, TRP1, HIS4-LEU2-(NBam;ori) (het),  rad50S::URA3, rad53::rad53-KD-(K227A)  rad50S::URA3, rad53::rad53-KD-(K227A) | This study |
| H6002 | MATa, ho::LYS2, ura3, LEU2, trp1::hisG, HIS3,  MATalpha, ho::LYS2, ura3, leu2::hisG, trp1::hisG, his3::hisG,  ddc2(lcd1)Δ::TRP1, sml1Δ::KanMX, rad50S::URA3  ddc2(lcd1)Δ::TRP1, sml1Δ::KanMX, rad50S::URA3 | This study |
| H6097 | MATa, ho::LYS2, lys2, ura3, LEU2, HIS3, TRP1,  MATalpha, ho::LYS2, lys2, ura3, leu2::hisG, his3::hisG, trp1::hisG,  rad50S::URA3  rad50S::URA3 | This study |
| H6146 | MATa, ho::LYS2, ura3, LEU2, trp1::hisG, HIS3,  MATalpha, ho::LYS2, ura3, leu2::hisG, TRP1, his3::hisG,  dbf4::dbf4-NLSdeltaN221, rad50S::URA3  dbf4::dbf4-NLSdeltaN221, rad50S::URA3 | This study |
| H6296 | MATalpha ho::LYS2, ura3, leu2::hisG, trp1::hisG, his3::hisG,  MATalpha ho::LYS2, ura3, LEU2, TRP1, HIS3,  rad50S::URA3, dbf4::dbf4-delta71-221  rad50S::URA3, dbf4::dbf4-delta71-221 | This study |
| H6813 | MATa, ho::LYS2, lys2, leu2::hisG, his3::hisG, ura3, TRP1,  MATalpha, ho::LYS2, lys2, leu2::hisG, his3::hisG, ura3, TRP1?,  his4X::LEU2-URA3, dmc1Δ::ARG4, sml1Δ::KanMX, rad53Δ::TRP1  his4X::LEU2-URA3, dmc1Δ::ARG4, sml1Δ::KanMX, rad53Δ::TRP1 | This study |
| H6814 | MATa, ho::LYS2, lys2, leu2::hisG, his3::hisG, ura3, trp1::hisG,  MATalpha, ho::LYS2, lys2, leu2::hisG, his3::hisG, ura3, trp1::hisG,  his4X::LEU2-URA3 (het),  dmc1Δ::ARG4, dbf4Δ::TRP1, Pdbf4::Pdbf4-dbf4-m25-LEU2  dmc1Δ::ARG4, dbf4Δ::TRP1, Pdbf4::Pdbf4-dbf4-m25-LEU2 | This study |
| H6815 | MATa, ho::LYS2, lys2, leu2::hisG, his3::hisG, ura3, trp1,  MATalpha, ho::LYS2, lys2, leu2::hisG, his3::hisG, ura3, TRP1,  his4X::LEU2-URA3, dmc1Δ::ARG4, sml1Δ::KanMX,  his4X::LEU2-URA3, dmc1Δ::ARG4, sml1Δ::KanMX,  rad53::rad53-KD-(K227A)  rad53::rad53-KD-(K227A) | This study |
| H7099 | MATa, ho::LYS2, lys2, URA3, LEU2, HIS3, TRP1,  MATalpha, ho::LYS2, lys2, ura3, leu2::hisG, his3::hisG, trp1::hisG, | This study |
| H7302 | MATa, ho::LYS2, lys2, ura3, LEU2, his3::hisG, trp1::hisG,  MATalpha, ho::LYS2, lys2, URA3, leu2::hisG, his3::hisG, trp1::hisG,  sml1Δ::KanMX, REC114-13MYC::HIS3, rad53Δ::TRP1  sml1Δ::KanMX, REC114-13MYC::HIS3, rad53Δ::TRP1 | This study |
| H7305 | MATa, ho::LYS2, lys2, ura3, LEU2, his3::hisG, trp1::hisG,  MATalpha, ho::LYS2, lys2, URA3, leu2::hisG, his3::hisG, trp1::hisG,  sml1Δ::KanMX, REC114-13MYC::HIS3, mec1Δ::TRP1  sml1Δ::KanMX, REC114-13MYC::HIS3, mec1Δ::TRP1 | This study |
| H7309 | MATa, ho::LYS2, ura3, LEU2, his3::hisG, trp1::hisG,  MATalpha, ho::LYS2, LYS2, ura3, leu2::hisG, HIS3, TRP1,  dbf4::NLS-DBF4, rad50S::URA3  dbf4::NLS-DBF4, rad50S::URA3 | This study |
| H7312 | MATa, ho::LYS2, ura3, leu2::hisG, his3::hisG, trp1::hisG,  MATalpha, ho::LYS2, ura3, leu2::hisG, his3::hisG, trp1::hisG,  ndt80Δ::TRP1, spo11-Y135F-HA-URA3. RAD50-6HA::HIS3  ndt80Δ::TRP1, spo11-Y135F-HA-URA3. RAD50-6HA::HIS3 | This study |
| H7320 | MATa, ho::LYS2, lys2, ura3, LEU2, his3::hisG, trp1::hisG  MATalpha, ho::LYS2, lys2, URA3, leu2::hisG, his3::hisG, trp1::hisG  sml1Δ::KanMX, MRE11-13MYC-HIS3MX6, rad53Δ::TRP1  sml1Δ::KanMX, MRE11-13MYC-HIS3MX6, rad53Δ::TRP1 | This study |
| H7323 | MATa, ho::LYS2, lys2, ura, LEU2, his3::hisG, trp1::hisG  MATalpha, ho::LYS2, lys2, URA3, leu2::hisG, his3::hisG, trp1::hisG  sml1Δ::KanMX, MRE11-13MYC-HIS3MX6, mec1Δ::TRP1  sml1Δ::KanMX, MRE11-13MYC-HIS3MX6, mec1Δ::TRP1 | This study |
| H7335 | MATa, ho::LYS2, ura3(Δsma-pst::hisG), LEU2, trp1::hisG,  MATalpha, ho::LYS2, LYS2, ura3, leu2::hisG, TRP1,  his4X::LEU2-(NgoMIV)-URA3 (het),  DBF4, rad50S::URA3  dbf4::dbf4-NLSdeltaN221, rad50S::URA3 | This study |
| H7401 | MATa, ho::LYS2, lys2, URA3, HIS3, TRP1, dbf4::dbf4-NLSdeltaN221  MATalpha, ho::LYS2, lys2, ura3, his3::hisG, trp1::hisG, DBF4 | This study |
| H7447 | MATa, ho::LYS2, lys2, leu2::hisG, ura3, trp1::hisG, his3::hisG,  MATalpha, ho::LYS2, lys2, leu2::hisG, ura3, trp1::hisG, his3::hisG,  ndt80Δ::TRP1, spo11-Y135F-HA-URA3, sml1Δ::KanMX  ndt80Δ::TRP1, spo11-Y135F-HA-URA3, sml1Δ::KanMX  cdc6::pSCC1-3HA-CDC6::KanMX6  cdc6::pSCC1-3HA-CDC6::KanMX6 | This study |
| H7468 | MATa, ho::LYS2, lys2, leu2::hisG, ura3, trp1::hisG, his3::hisG,  MATalpha, ho::LYS2, lys2, leu2::hisG, ura3, trp1::hisG, his3::hisG,  ndt80Δ::TRP1, spo11-Y135F-HA-URA3  ndt80Δ::TRP1, spo11-Y135F-HA-URA3 | This study |
| H7469 | MATa, ho::LYS2, lys2, leu2::hisG, ura3, trp1::hisG, his3::hisG,  MATalpha, ho::LYS2, lys2, leu2::hisG, ura3, trp1::hisG, his3::hisG,  ndt80Δ::TRP1, spo11-Y135F-HA-URA3, DBF4  ndt80Δ::TRP1, spo11-Y135F-HA-URA3, dbf4::dbf4-NLSdeltaN221 | This study |
| H7493 | MATa, ho::LYS2, lys2, leu2::hisG, ura3, trp1::hisG, his3::hisG,  MATalpha, ho::LYS2, lys2, leu2::hisG, ura3, trp1::hisG, his3::hisG,  ndt80Δ::TRP1, DBF4  ndt80Δ::TRP1, dbf4::dbf4-NLSdeltaN221 | This study |
| H7494 | MATa, ho::LYS2, lys2, leu2::hisG, ura3, trp1::hisG, his3::hisG,  MATalpha, ho::LYS2, lys2, leu2::hisG, ura3, trp1::hisG, his3::hisG,  ndt80Δ::TRP1  ndt80Δ::TRP1 | This study |

**References:**

Bishop DK, Park D, Xu L, Kleckner N. DMC1: a meiosis-specific yeast homolog of E. coli recA required for recombination, synaptonemal complex formation, and cell cycle progression. 1992. *Cell* **3**:439-56. doi: 10.1016/0092-8674(92)90446-J.

Blitzblau HG, Chan CS, Hochwagen A, Bell SP. Separation of DNA replication from the assembly of break-competent meiotic chromosomes. 2012. *PLoS Genet* **5**:e1002643.

doi: 10.1371/journal.pgen.1002643.

Hochwagen A, Tham WH, Brar GA, Amon A. The FK506 binding protein Fpr3 counteracts protein phosphatase 1 to maintain meiotic recombination checkpoint activity. 2005. *Cell* **6**:861-73. doi: 10.1016/j.cell.2005.07.010.
